# Genomic and virologic characterization of samples from a shipboard outbreak of COVID-19 reveals distinct variants within limited temporospatial parameters

**DOI:** 10.3389/fmicb.2022.960932

**Published:** 2022-08-10

**Authors:** Regina Z. Cer, Logan J. Voegtly, Bishwo N. Adhikari, Brian L. Pike, Matthew R. Lueder, Lindsay A. Glang, Francisco Malagon, Ernesto Santa Ana, James M. Regeimbal, Maria F. Potts-Szoke, Kevin L. Schully, Darci R. Smith, Kimberly A. Bishop-Lilly

**Affiliations:** ^1^Department of Genomics and Bioinformatics, Biological Defense Research Directorate, Naval Medical Research Center, Fort Detrick, MD, United States; ^2^Leidos, Reston, VA, United States; ^3^Defense Threat Reduction Agency, Ft. Belvoir, VA, United States; ^4^Department of Operations, Biological Defense Research Directorate, Naval Medical Research Center, Fort Detrick, MD, United States; ^5^The Austere Environments Consortium for Enhanced Sepsis Outcomes (ACESO), Biological Defense Research Directorate, Naval Medical Research Center, Fort Detrick, MD, United States; ^6^Department of Microbiology and Immunology, Biological Defense Research Directorate, Naval Medical Research Center, Fort Detrick, MD, United States

**Keywords:** shipboard outbreak, COVID-19, genomic characterization, aircraft carrier, molecular epidemiology

## Abstract

Early in the pandemic, in March of 2020, an outbreak of COVID-19 occurred aboard the aircraft carrier USS Theodore Roosevelt (CVN-71), during deployment in the Western Pacific. Out of the crew of 4,779 personnel, 1,331 service members were suspected or confirmed to be infected with SARS-CoV-2. The demographic, epidemiologic, and laboratory findings of service members from subsequent investigations have characterized the outbreak as widespread transmission of virus with relatively mild symptoms and asymptomatic infection among mostly young healthy adults. At the time, there was no available vaccination against COVID-19 and there was very limited knowledge regarding SARS-CoV-2 mutation, dispersal, and transmission patterns among service members in a shipboard environment. Since that time, other shipboard outbreaks from which data can be extracted have occurred, but these later shipboard outbreaks have occurred largely in settings where the majority of the crew were vaccinated, thereby limiting spread of the virus, shortening duration of the outbreaks, and minimizing evolution of the virus within those close quarters settings. On the other hand, since the outbreak on the CVN-71 occurred prior to widespread vaccination, it continued over the course of roughly two months, infecting more than 25% of the crew. In order to better understand genetic variability and potential transmission dynamics of COVID-19 in a shipboard environment of immunologically naïve, healthy individuals, we performed whole-genome sequencing and virus culture from eighteen COVID-19-positive swabs collected over the course of one week. Using the unique variants identified in those genomes, we detected seven discrete groups of individuals within the population aboard CVN-71 infected with viruses of distinct genomic signature. This is in stark contrast to a recent outbreak aboard another U.S. Navy ship with >98% vaccinated crew after a port visit in Reykjavik, Iceland, where the outbreak lasted only approximately 2 weeks and the virus was clonal. Taken together, these results demonstrate the utility of sequencing from complex clinical samples for molecular epidemiology and they also suggest that a high rate of vaccination among a population in close communities may greatly reduce spread, thereby restricting evolution of the virus.

## Introduction

COVID-19, which is caused by severe acute respiratory syndrome coronavirus 2 (SARS-CoV-2), has emerged as a public health crisis all over the world. Early in the pandemic, in March of 2020, an outbreak of COVID-19 occurred aboard the aircraft carrier USS Theodore Roosevelt during deployment to the Western Pacific. Out of 4,779 ship crew personnel, 1,331 service members (27.9%) were suspected or confirmed to be infected with SARS-CoV-2 (Alvarado et al., 2020; Kasper et al., 2020). Subsequent investigations into this outbreak have characterized it as widespread transmission of virus with relatively mild symptoms and asymptomatic infection among mostly young and healthy adults (Alvarado et al., 2020; Kasper et al., 2020; Payne et al., 2020). This was significant because at the time of this outbreak, data from SARS-CoV-2 viral infections were primarily ascertained from older and immunocompromised components of the population, and there was not much information from outbreaks among younger, healthy populations living in close quarters. Additionally, at that time, vaccination against COVID-19, a key element to limiting the spread of SARS-CoV-2, was not available. Since then, there have been documented studies of SARS-CoV-2 transmission dynamics in the close quarter settings that are characteristic of the U.S. military (Letizia et al., 2020, 2021a,b). To understand how the SARS-CoV-2 virus is transmitted, mutated, and dispersed among unvaccinated service members in a shipboard environment, this retroactive study examines viral characterization data from nasal swab samples obtained from 18 individuals with confirmed or suspected infection. Analysis of these SARS-CoV-2 genomes demonstrates the existence of multiple genetic variants of the virus within the same population over a very short length of time.

The shipboard environment is a unique and challenging environment, in a number of ways, particularly when it comes to infectious disease control. Although it has been 2 years since the pandemic began, there are very limited genomic analyses performed on viral samples from shipboard outbreaks in general, including both military and commercial vessels. Literature searches were performed in PubMed, the National Center of Biotechnology Information, and Google Scholar for articles published in English between 2019 and April 25, 2022, with keyword combination of “SARS-CoV-2” or “genomic characterization” and “ship.” This search resulted in a few COVID-19 outbreak reports on the Diamond Princess cruise ship in Japan in February 2020 (Sekizuka et al., 2020; Yeh and Contreras, 2021), a fishing vessel that departed from Seattle, Washington, in May 2020 with 122 COVID cases (Addetia et al., 2020), the USNS COMFORT which was deployed in New York City to assist the inpatient health care capacity in New York City and later had 13 cases arise onboard (Lalani et al., 2021), the USS Ronald Reagan (CVN-76; Mullinax et al., 2022), a Navy vessel off the coast of Iceland (Servies et al., 2022), as well as follow-up reviews of some of these outbreaks (Batista et al., 2020; Vicente et al., 2021). These studies, however, mainly focused on demographic, epidemiologic, and general phenotypic characterization of COVID positive samples and hardly on genomic characterization of the pathogen itself. Fortunately, not every vessel will experience an outbreak, as has been demonstrated by the USS Harry S. Truman Strike Group deployed from Norfolk, VA, in November 2019, where there was no outbreak and the ship returned to its home port in June 2020 with zero COVID-19 cases (Bigornia, 2021), but understanding how a novel pathogen may spread and diverge in such an environment may help to develop effective countermeasure strategies.

A limited number of studies of shipboard outbreaks with viral genetic analyses conducted exist, but these include: the Diamond Princess Cruise ship study that analyzed SARS-CoV-2 genomes from 28 individuals, the fish vessel outbreak study with 39 genomes sequenced out of 122 individuals infected, and the Navy vessel off the coast of Iceland, from which 18 samples were sequenced. Widespread vaccination and high rates of natural infection have dramatically limited our ability to examine the natural history of SARS-CoV-2 transmission and mutation within an immunologically naïve population in very close quarters. Here, we present a retrospective study that is the first report of the Theodore Roosevelt (CVN-71) outbreak that includes the application of viral genome sequencing with virologic and epidemiological data to study pathogen variations arising within an immunologically naïve population confined to close quarters. Despite the outbreak occurring in an isolated shipboard environment where it might be expected that one viral strain might rapidly multiply, we identified distinct variations occurring within the cohort in a very short timeframe. Based on some unique genetic variations identified in those genomes, we were able to categorize the subjects into seven groups with viruses of distinct genomic signatures that build upon one another as sequential mutations in a very short time, demonstrating possible transmission chains. The implications of constant and rapid evolution of SARS-CoV-2 could be relevant in efforts to halt transmission chains and to enable a more targeted approach to disease control in a shipboard environment for this, and potential future, pandemics.

## Materials and methods

### Study design, sample collection, and RT-PCR

**Figure 1:**
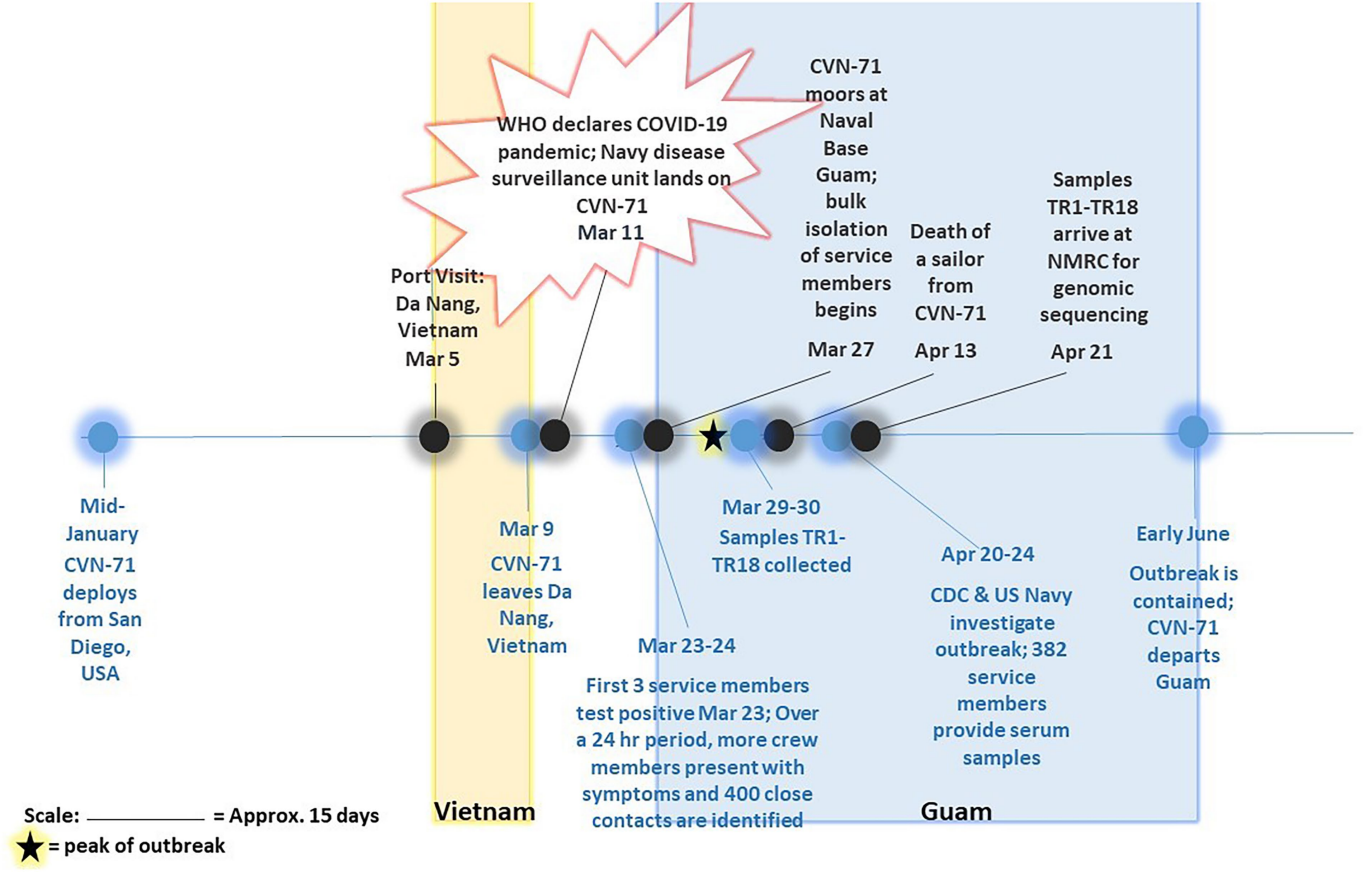
Timeline of COVID-19 outbreak on aircraft carrier USS Theodore Roosevelt in 2020. Major events pertaining to and surrounding outbreak of COVID-19 on CVN-71 as gathered from available references (Alvarado et al., 2020; Payne et al., 2020; Pike and Koblentz, 2020; Stewart and Ali, 2020; U.T.R.P. Affairs, 2020) are depicted, including collection of 18 samples of convenience for sequencing-based surveillance. The peak of outbreak as per Alvarado et al. (2020) is indicated on the timeline with a star.

Eighteen SARS-CoV-2-positive samples from the COVID-19 outbreak aboard the USS Theodore Roosevelt (CVN-71) were samples of convenience that were randomly selected from symptomatic individuals that presented to sick call aboard the ship on March 29 and March 30, 2020 (Figure 1), de-identified, and sent for viral culture and genome sequencing. These samples were collected as part of the ship’s outbreak response, which involved testing of suspected COVID-19 cases and close contacts as previously reported (Kasper et al., 2020). Briefly, nasopharyngeal (NP) swab specimens were collected from individuals using viral transport medium (VTM) swab kits. Samples were processed with either the Qiagen QIAamp Viral RNA Mini Kit or the Roche MagNA Pure 96 instrument for automated nucleic acid extraction per the manufacturer’s instructions. The presence of SARS-CoV-2 infection was determined by the Seegene Allplex 2019-nCOV assay test kit (Seegene Technologies) or by the Centers for Disease Control and Prevention (CDC) emergency-use-authorization (EUA) assay, each using primers targeting two sites in the nucleocapsid gene, N1 and N2.

### Virus culture

The viral component of each sample was cultured under Biosafety Level 3 (BSL-3) conditions and simultaneously sequenced from primary material under nonhuman subject research determination PJT 20–08. Patient samples were cultured for SARS-CoV-2 using a standard plaque assay on Vero cells in six-well plates as well as by a cytopathic effect (CPE) assay on Vero cells in T-25 cm^2^ flasks. For the plaque assay, duplicate wells were infected with 0.2 ml aliquots from a 1:2 and serial 10-fold dilutions in Minimum Essential Medium (MEM), followed by an hour incubation at 37°C with 5% CO_2_ to allow virus adsorption to occur. After incubation, cells were overlaid with MEM containing 0.5% agar supplemented with 5% heat-inactivated fetal bovine serum (FBS) and 1% penicillin/streptomycin and incubated for 72 h at 37°C with 5% CO_2_. Cells were fixed in 10% formalin prior to staining with crystal violet and plaque counting. For the CPE assay, cells were seeded in T-25 cm^2^ flasks and each flask was infected with 0.5 ml aliquots from a 1:2, 1:5, and 1:10 dilution in MEM, followed by a 1 h incubation at 37°C with 5% CO_2_. After incubation, 5 ml of MEM was supplemented with 5% heat-inactivated FBS, 1% penicillin/streptomycin was added, and the flasks were incubated for 5 days at 37°C with 5% CO_2_. CPE was monitored daily and post 5 days, supernatant was passed onto fresh cells to allow additional time to amplify.

### Library preparation and genome sequencing

RNA was extracted from 0.25 ml of VTM using 0.75 ml of TRIzol LS reagent (Invitrogen) according to the manufacturer’s protocol. RNA concentration was measured using Qubit RNA High-Sensitivity assay (Thermo Fisher Scientific) prior to use in the ARTIC v3 nCoV-2019 Sequencing protocol (Quick, 2020) with the exception of one sample (i.e., TR1) that required additional sequencing, for which the YouSeq v2 SARS-CoV-2 Coronavirus NGS Library preparation kit was used. In that case, the YouSeq reverse transcriptase was replaced with SuperScript IV (Thermo Fisher Scientific). Complementary DNA (cDNA) was amplified using multiplex PCR and either the associated ARTIC primer pools or YouSeq primer pools. Samples prepared *via* the ARTIC protocol were cleaned using 1x AMPure XP beads (Beckman Coulter) and resuspended in nuclease-free molecular grade water. Sequencing libraries were completed following the QiaSeq FX protocol (Qiagen). Libraries were checked for quality using an Agilent Bioanalyzer High-sensitivity kit (Agilent) and quantitated using the Qubit DNA High-Sensitivity assay (Thermo Fisher Scientific) prior to sequencing using Illumina MiSeq v3 2×300 chemistry (Illumina).

### Bioinformatic analyses

Raw sequencing reads were processed using Viral Amplicon Illumina Workflow (VAIW, version 1; Batista et al., 2020). Briefly, the reads were first assessed for quality (Q20) and trimmed using bbduk.^1^ Then, resulting paired reads were merged using bbmerge and aligned to the Wuhan reference genome (NC_045512.2) using bbmap (Bushnell, 2014). ARTIC or YouSeq primers were trimmed from sequence ends using align_trim (ARTIC pipeline). The consensus genome was generated and Single-Nucleotide Variants (SNVs) were determined using samtools mpileup (Li et al., 2009) and iVar (Intrahost Variant Analysis of Replicates; Grubaugh et al., 2019) with a minimum coverage of 10x and minimum nucleotide frequency of 30%. Global lineage was determined using Phylogenetic Assignment of Named Global Outbreak Lineages (PANGOLin v3.1.19). A maximum-likelihood tree was generated using 16 representative sequences, the Wuhan Hu-1 reference, and the 18 samples collected for this study (herein called TR samples) using MAFFT (Katoh et al., 2002) and IQ-tree 2 Ml (GTR + G; Minh et al., 2020). Resulting trees were visualized using FigTree (Rambaut, 2012). The 16 representative sequences were obtained by selecting a subset of B.1.1 reference genomes from Global Initiative on Sharing All Influenza Data repository (GISAID accessed, April 8, 2022) by filtering for genome completeness, high coverage, and collection date availability up to April 1, 2020, to match the timing of the sample collection. These samples were further reduced by removing sequences that contain stretches of ambiguous or unsequenced bases (represented as “N” in reference sequences) and by clustering with mmseqs (v13.45111; Hauser et al., 2016) to remove duplicates. The remaining samples were processed through nextclade (v10.0.10) to identify SNVs present in the samples. Final representatives, which had the earliest collection date and the same core SNVs as the TR samples, were chosen, thereby reducing the dataset from 5,730 samples to 16 samples, representing 1,415 identical samples.

## Results

### PCR testing

Eighteen COVID-positive surveillance samples of convenience from patients who presented to sick call aboard the Theodore Roosevelt over a 2-day period between March 29 and 30, 2020, at the peak of the outbreak (Alvarado et al., 2020; Figure 1) were sent for viral isolation and sequencing (Table 1). Three samples, TR16, TR17, and TR18, were initially identified as close contacts of other cases on the ship and subsequently tested within a pooled testing format and found negative on March 24 or March 25, 2020. However, when tested again on March 30, 2020, they became COVID positive. Most of the cases had very low real-time PCR cycle threshold (CT value ~10) to relatively low CT values (<20) indicating high viral loads with the exceptions of two samples: TR1 (N1: 26.69 and N2: 30.06) and TR3 (N1: 31.32 and N2: 35.11), for which PCR test result for the latter was indeterminate.

**Table 1:** Metadata of samples included in study.

Sample ID	Referral source	Sample collection date	COVID-19 test		Ct value		
			Date	Result	N1	N2	RNaseP
TR1	Sick call	3/30/2020	3/30/2020	Positive	26.69	30.06	19.28
TR2	Sick call	3/30/2020	3/30/2020	Positive	23.23	25.3	20.28
TR3	Sick call	3/29/2020	3/30/2020	Indeterminate	31.32	35.11	24.35
TR4	Sick call	3/30/2020	3/30/2020	Positive	15.53	15.55	26
TR5	Sick call	3/30/2020	3/30/2020	Positive	UND	13.2	24.14
TR6	Sick call	3/30/2020	3/30/2020	Positive	19.59	19.82	23.55
TR7	Missing data	3/29/2020	3/30/2020	Positive	20.02	21.74	18.54
TR8	Sick call	3/30/2020	3/30/2020	Positive	10	9.13	22.31
TR9	Sick call	3/30/2020	3/30/2020	Positive	23.96	24.12	24.69
TR10	Sick call	3/30/2020	3/30/2020	Positive	UND	12	19.17
TR11	Sick call	3/30/2020	3/30/2020	Positive	20.36	22.53	24.21
TR12	Sick call	3/29/2020	3/30/2020	Positive	UND	12.13	24.39
TR13	Sick call	3/30/2020	3/30/2020	Positive	18.69	19.01	22.72
TR14	Sick call	3/30/2020	3/30/2020	Positive	16.34	17.65	22.62
TR15	Sick call	3/30/2020	3/30/2020	Positive	10.1	10.4	22.26
TR16^*^	Close contacts	3/24/2020	3/24/2020	Negative	N/A (pooled)	N/A (pooled)	
	Sick call	3/30/2020	3/30/2020	Positive	14.5	15.57	25.42
TR17^*^	Close contacts	3/24/2020	3/25/2020	Negative	N/A (pooled)	N/A (pooled)	
	Sick call	3/30/2020	3/30/2020	Positive	16.7	19.12	25.25
TR18^*^	Close contacts	3/25/2020	3/26/2020	Negative	N/A (pooled)	N/A (pooled)	
	Sick call	3/30/2020	3/30/2020	Positive	18.67	20.31	24.55

N/A = not applicable; UND = undetermined;

* Identified as close contacts of other cases on the ship and thus tested within a pooled testing format and found negative 5–6 days prior before becoming positive on March 30, 2020.

### Virus culture

Aliquots of VTM from each individual were assessed by CPE assay on Vero cells in T-25 cm^2^ flasks that were monitored for signs of growth and by standard plaque assay in six-well plates to determine the infectious virus titer. Three samples, TR1, TR11, and TR13, were negative by both CPE and plaque assays. Two samples, TR2 and TR9, were positive for CPE but negative by plaque assay. The remaining 13 samples were positive by both CPE and plaque (with titers ranging from 2.9 to 5.3 log_10_ plaque-forming units per ml PFU/ml) assays (Table 2). Sample TR3, which was “indeterminate” by PCR assay, was determined to have a midrange titer of 4.5 log_10_ PFU/ml in the plaque assay. This suggests that other factors than low titer may have caused the indeterminate PCR result and that indeterminate PCR results may still merit genome sequencing in an outbreak investigation, particularly if taken from a symptomatic patient and/or a patient with epidemiology info such as a known exposure.

**Table 2:** Virus cultivation results and genome sequencing statistics.

Sample ID	Titer (PFU/ml)	Cytopathic effect (CPE)	Number of raw sequencing reads	Consensus genome length (bp)	Virus lineage/nextStrain clade/GISAID clade
TR1	Negative	Negative	5,606,273	29,801	B.1.1/20B/GR
TR2	Negative	Positive	7,792,070	29,782	B.1.1/20B/GR
TR3	4.5 log_10_	Positive	9,483,672	29,782	B.1.1/20B/GR
TR4	4.4 log_10_	Positive	2,217,062	29,782	B.1.1/20B/GR
TR5	5.3 log_10_	Positive	6,332,426	29,782	B.1.1/20B/GR
TR6	3.6 log_10_	Positive	7,750,816	29,782	B.1.1/20B/GR
TR7	2.9 log_10_	Positive	1,327,290	29,782	B.1.1/20B/GR
TR8	4.5 log_10_	Positive	4,580,614	29,782	B.1.1/20B/GR
TR9	Negative	Positive	8,195,212	29,782	B.1.1/20B/GR
TR10	3.7 log_10_	Positive	4,096,140	29,782	B.1.1/20B/GR
TR11	Negative	Negative	4,375,622	29,782	B.1.1/20B/GR
TR12	4.1 log_10_	Positive	2,102,416	29,782	B.1.1/20B/GR
TR13	Negative	Negative	3,039,242	29,782	B.1.1/20B/GR
TR14	3.6 log_10_	Positive	10,141,716	29,782	B.1.1/20B/GR
TR15	5.3 log_10_	Positive	3,130,260	29,782	B.1.1/20B/GR
TR16	3.4 log_10_	Positive	1,316,262	29,782	B.1.1/20B/GR
TR17	3.5 log_10_	Positive	6,894,320	29,782	B.1.1/20B/GR
TR18	5.3 log_10_	Positive	3,130,260	29,782	B.1.1/20B/GR

### Genome sequencing and assembly

Despite differences in Ct values and viral titers, including some samples that did not grow in cell culture at all, coding complete viral genomes were achieved from all eighteen samples, indicating that even if the samples did not contain viable virus any longer, they did contain sufficient viral nucleic acids. Sample TR1, which had higher Ct values (N1: 26.69, N2:30.06) than TR11 and TR13 negative by both assays, was sequenced using two different and complementary SARS-CoV-2 amplicon sequencing strategies, ARTIC and YouSeq. The additional sequencing protocol applied to this sample generated more sequence data from the 5′ and 3′ noncoding regions than for other samples, resulting in a slightly longer consensus genome length of 29,801 nt as opposed to 29,782 nt in other samples. Another sample with a higher CT value, sample TR3 (N1:31.32, N2: 35.11), required multiple library preparations to yield a full viral genome.

**Figure 2:**
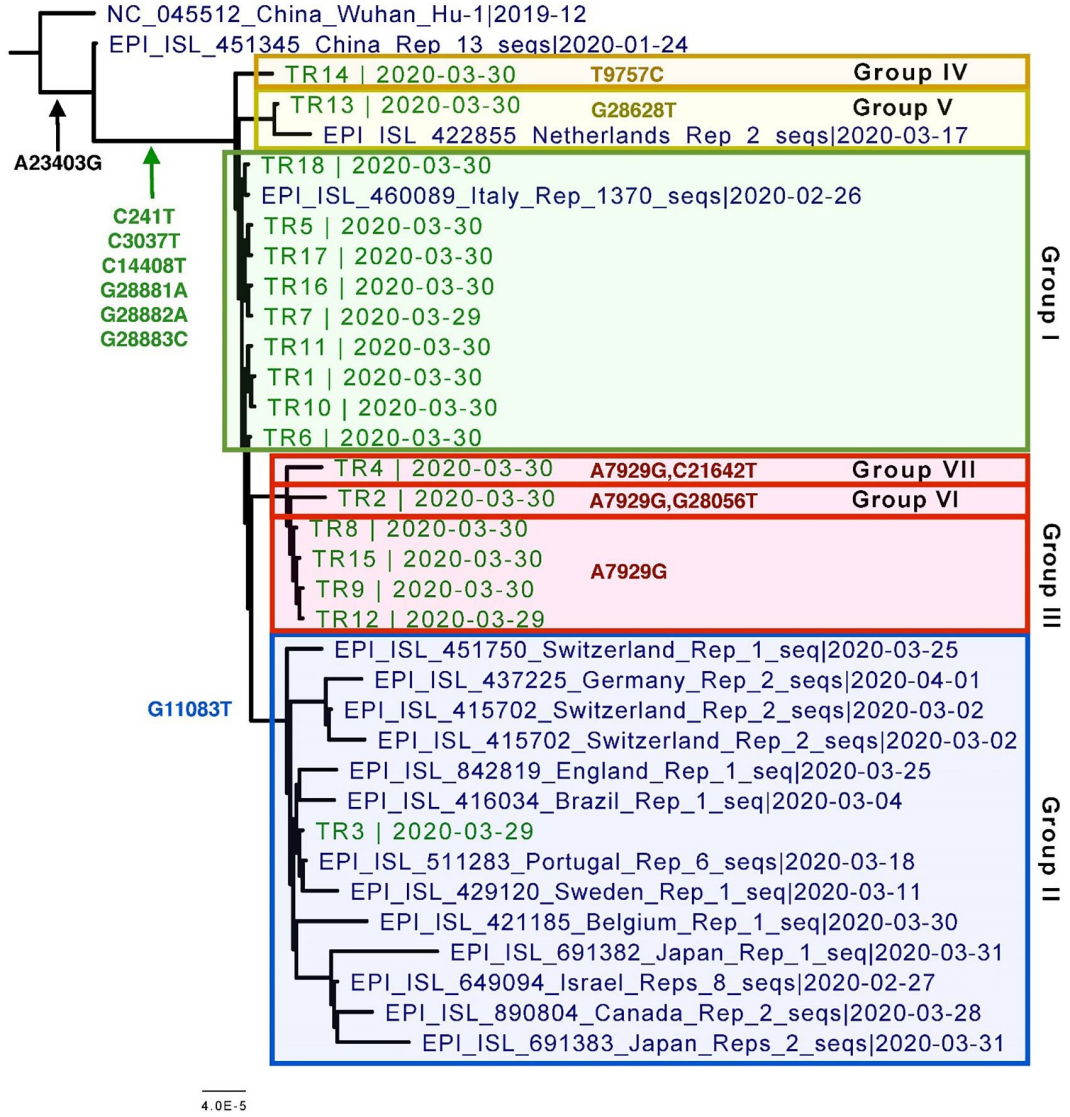
Phylogenetic tree of the eighteen CVN-71 viral genomes in relation to representative samples from lineages circulating during the beginning of the pandemic in early 2020. Based on the SNVs, groupings are shown in different colored boxes. Except for the two earliest references from China, which are B and B.1, the rest of the sequences included in the tree are B.1.1 lineages.

Overall, all of the 18 genomes were very similar to each other; all of them belonged to Pangolin lineage B.1.1, NextStrain clade 20B, and GISAID clade GR (Table 2; Figure 2). They all contained mutations: A23403G (D614G), C241T (5’UTR), C3037T (F924F), C14408T (P4715L), G2881A (R203K), G28882A (R203R), and G28883C (G204R). This is in contrast to one specific report on viral genome sequence analysis from cases on the Diamond Princess, in which 8 of 28 cases were identical to the original Wuhan WIV-4 sequence (Yeh and Contreras, 2021). This constellation of multiple variations in common among the samples is not surprising since the samples were all collected within 2 days and from a closely knit community. Based on the phylogenetic tree placement, viral sequences from 18 samples can be categorized into seven discrete groups. Group I consisted of genomes from the three samples (TR16, TR17, and TR18) which were collected from close contacts and tested negative initially on March 24 and March 25. Group 1 also included viruses from samples TR1 and TR11, which were negative by both CPE and plaque assays. Sample TR3, which had the highest CT value among the 18 samples with indeterminate PCR result, produced the only genome belonging to Group II. Genomes from samples TR2 and TR9, which were positive samples for CPE but too low titer to quantify, belonged to Groups VI and III, respectively. Finally, sample TR13 that was negative by both CPE and titer assays produced a genome that belonged to Group V.

**Figure 3:**
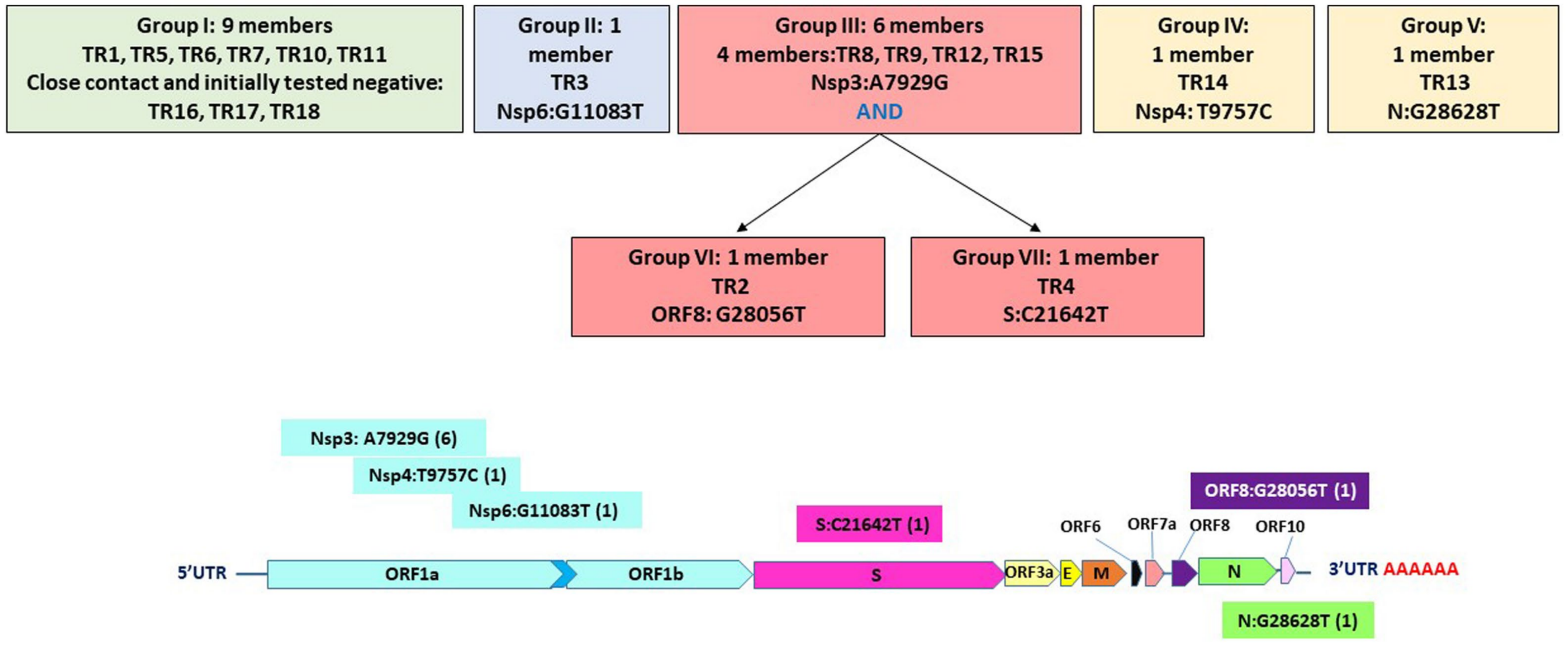
Seven groupings based on six high-quality single-nucleotide variations and their positions on the genome.

Specifically, these groups are based on the presence or absence of six high-quality single-nucleotide variations (SNVs): A7929G (K2555R in ORF1a), R3164R (T9757C in ORF1a), L37F (G11083T in ORF1a), A27V (C21642T in S), A55S (G28056T in ORF8), and A119S (G28628T in N; Figure 3; Table 3). These six SNVs are not any of the five characteristic mutations of B.1.1: P314L (ORF1b), D614G (S), S84L (ORF8), R203K (N), and G204R (N). Briefly, one sample each was found to have a non-synonymous SNV in the S gene that encodes spike glycoprotein (TR4), ORF8 (TR2), or the nucleocapsid phosphoprotein gene (TR13). Notably, the A7929G SNV in nsp3 that results in a non-synonymous mutation K2555R was shared among 33% of the samples (n = 6, TR2, TR4, TR8, TR9, TR12, and TR14) and this was the only SNV found in common with any other SNV in a single genome. This is in contrast with the findings from the Diamond Princess cruise ship study where the G11083T SNV in nsp6 that results in an L37F mutation was observed in 14% (4/28) of the samples spread during shipboard quarantine and arose through *de novo* RNA recombination under positive selection pressure (Yeh and Contreras, 2021). In the Theodore Roosevelt (CVN-71) shipboard outbreak, we observed L37F in only 5% (1/18) of the group. It is important to note that the K2555R mutation in ORF1a appears to be unique to the Theodore Roosevelt shipboard outbreak as compared to other published B.1.1 genomes from that period.

**Table 3:** Single nucleotide variations and effects.

Variant	# of samples	Group	Coverage^§^	Allele frequency (%)	Gene	Protein product affected	synonymous/nonsynonymous	Amino acid change	Biological significance
A7929G	6	III, VI, VII	8495^£^	99.7^£^	ORF1a	nsp3	Non-synonymous	K2555R	No report found
T9757C	1	IV	15913	98.2	ORF1a	nsp4	synonymous	Not applicable	Not applicable
G11083T	1	II	17012	99.9	ORF1a	nsp6	Non-synonymous	L37F	Associated with asymptomatic SARS-CoV-2 infection. Linked to viral hypotoxicity (Wang et al., 2020; Sun et al., 2022)
C21642T	1	VII	7117	99.9	S	spike	Non-synonymous	A27V	Located on N-terminal domain; no significant effects (Ferrareze et al., 2021)
G28056T	1	VI	5222	95.4	ORF8	ORF8 protein	Non-synonymous	A55S	Destabilizes the RNA binding domain (Rahman et al., 2021)
G28628T	1	V	6174	100	N	Nucleocapsid phosphoprotein	Non-synonymous	A119S	N:A119S is one of the five lineage-defining SNVs that distinguish P.2 (B.1.1.28.2) sequences from all other B.1.1.28 sequences available in Brazil (Resende et al., 2021)

N/A = not applicable.

§ Number of reads supporting alternative (mutant) allele.

£ Average of six samples with read coverage (3253, 5954, 7559, 9790, 11566, 12847).

**Figure 4:**
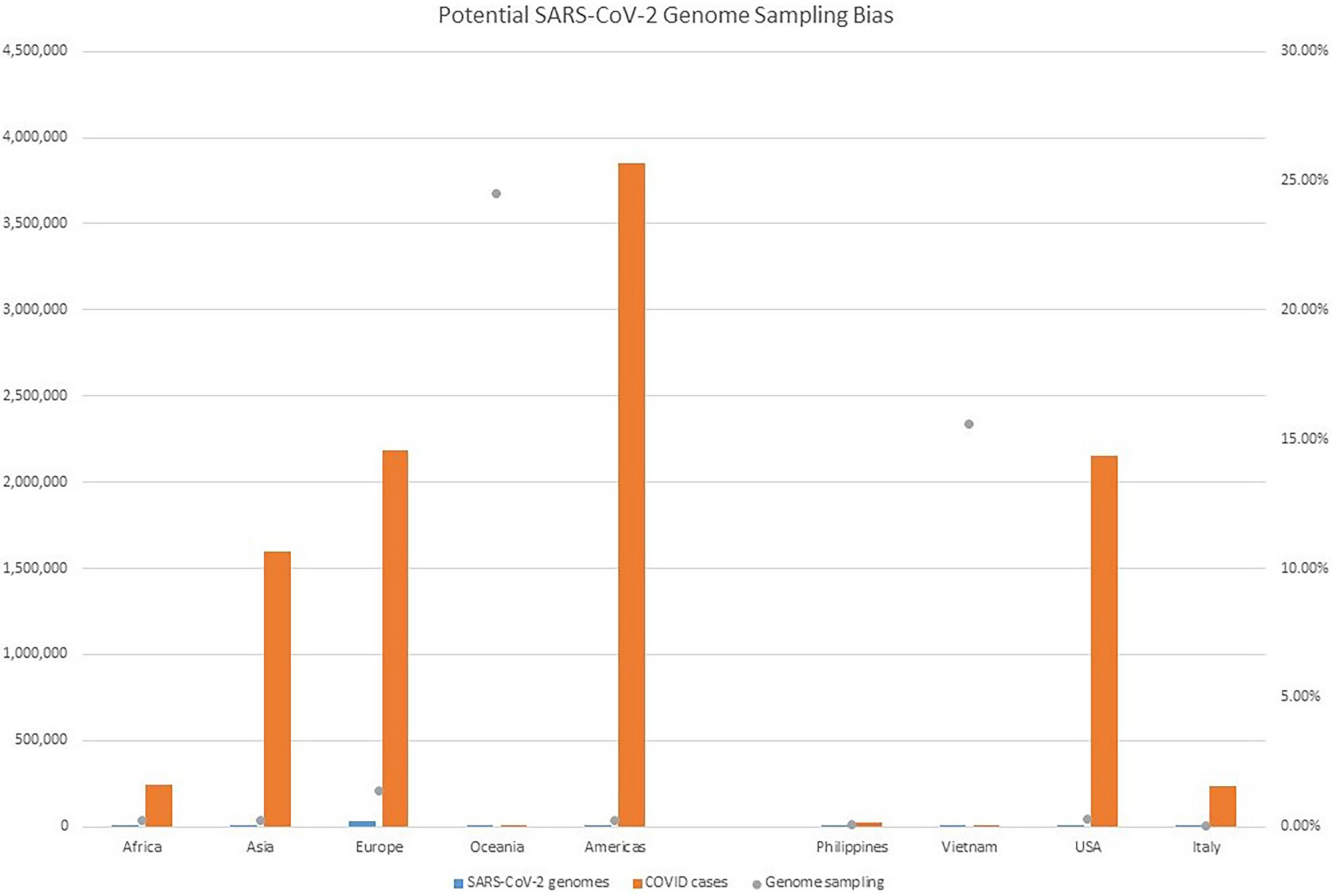
Potential SARS-CoV-2 Genome Sampling Bias. The number of cases per geographic region at the time of the outbreak aboard CVN-71 is represented by red bars and the left *y* axis, the number of viral genomes in GISAID for each geographic region at the time is depicted by blue bars and the left *y* axis, and is depicted by blue bars and the left *y* axis, and the proportion of viral genomes sampled per geographic region is denoted with a gray dot. Number of cases by continent was taken from https://www.ecdc.europa.eu/en/geographical-distribution-2019-ncov-cases (accessed on June 12, 2020).

Despite all these distinct genomic signatures, sample bias in data availability from different parts of the world was an inevitable issue that made the confident assignment of the geographic origin of the outbreak impossible. In this case, B.1.1 lineage reference genomes from samples collected through April 1, 2020, in GISAID were mainly from Europe (4,058), followed by North America (625), Asia (580), Oceania (284), South America (131), and Africa (25). We found certain parts of Asia to be undersampled in SARS-CoV-2 genome sequencing. For instance, at the time of these genomes being sequenced, specifically looking at GISAID submission dates through June 12, 2020, to allow time for sample processing and submission post sample collection, we found that there were 46,636 SARS-CoV-2 genomes available in GISAID, without filtering, and of those, only 3,875 (8.31%) were from Asia. If we applied filtering for complete genomes and low depth of coverage, 31,729 total genomes were available, of that, 2,796 (8.81%) were from Asia. In other words, whether with or without filtering, the entire continent of Asia was contributing less than 10% of the reference genomes for SARS-CoV-2 worldwide, despite the outbreak having been observed there first (Table 4) and there being ~20% of COVID cases worldwide from that area, demonstrating strong biases toward sequencing from samples taken in Europe and Oceania in general. Specifically analyzing data by country indicates undersampling in the Philippines and oversampling in Vietnam. That is, within Asia, there were only 17 reference SARS-CoV-2 genomes available in GISAID for the Philippines although it reported 26,420 COVID cases (i.e., 0.06%), whereas Vietnam submitted 48 genomes (14.37%) out of 334 COVID cases reported at the time (Figure 4). Due to this unevenness in viral genome sampling from different geographic regions around the world at that time and the possibility that the databases might therefore lack enough representative sequences for a given region, speculation on the origin of the outbreak based on sequencing-based surveillance on the Theodore Roosevelt (CVN-71) was not attempted.

**Table 4:** SARS-CoV-2 reference genomes and cases by geographic region at the time of viral genome sequencing from Theodore Roosevelt outbreak (up to June 12, 2020).

			GenBank/NCBI		GISAID	
	Geographic region	Total number of COVID cases	Total number of SARS-CoV-2 genomes	Genome sampling %	Total number of SARS-CoV-2 genomes	Genome sampling %
By continent	Africa	243,125	539	0.22	469	0.19
	Asia	1,594,208	3,780	0.24	3,875	0.24
	Europe	2,187,307	29,911	1.37	29,980	1.37
	Oceania	8,796	2,157	24.52	2,152	24.47
	Americas	3,848,098	10,149	0.26	10,160	0.26
By country	Philippines	26,420	17	0.06	17	0.06
	Vietnam	334	52	15.57	48	14.37
	United States	2,150,000	5,973	0.28	8,688	0.40
	Italy	237,000	108	0.05	147	0.06

## Discussion

Herein, we have presented a unique genetic and virologic investigation of a shipboard outbreak of an emerging viral pathogen. The 18 viral genomes from servicemen and servicewomen onboard the Theodore Roosevelt (CVN-71) were sequenced from samples taken in a span of 2 days only approximately 1 week post recognition of the outbreak on the ship, and represented only a small fraction of the total cases. Yet, they contained variations that allowed them to be separated into seven distinct groups. One particular variant, A7929G, a non-synonymous variation in nsp3, was favored among this sample set occurring in 33% of the cohort. Nsp3 is an essential component of SARS-CoV-2 replication (Lei et al., 2018) and is considered a potential target for antiviral drugs (Baez-Santos et al., 2015). It is interesting to note that despite the close timeline of outbreaks between Diamond Princess Cruise ship (February 10–25, 2020) and the Theodore Roosevelt ship outbreak (March 24, 2020), the most common mutation differed between them: L37F and K2555R being the most common, respectively. In fact, it is interesting to note that the K255R mutation appears to be unique to the Theodore Roosevelt shipboard outbreak. In addition, we have demonstrated a sampling bias in terms of viral genome sequencing in different regions around the world at the time, a bias that would somewhat limit conclusions that could be drawn regarding the potential source of infection. It is important to remember that these are potentially important caveats to the interpretation of data regarding predominance of a given lineage over another in certain geographic regions, as well as the origin of the virus in this particular shipboard outbreak.

Recently, sequencing-based surveillance was applied to another Navy shipboard outbreak of COVID-19, and the findings were rather different in that more recent study, there was much less genetic variation observed. In fact, in that more recent study, of the 18 samples sequenced, 16 samples were 100% identical to each other. The genome of the 17th sample was found to be only one nucleotide different from the sixteen samples and the 18th sample did not produce a sufficient consensus genome to be analyzed in this manner (Servies et al., 2022). The most salient difference between these two outbreaks is that the outbreak on CVN-71 occurred prior, in an unvaccinated crew, whereas the other outbreak occurred more recently among a highly vaccinated crew. Taken together, these results suggest that a high rate of vaccination among a population in close quarters may greatly reduce spread, thereby restricting evolution of the virus; an important reminder for future pandemics. In fact, a recent analysis published online prior to peer review investigates the potential relationship between vaccination rates in various countries as compared to viral mutation rate, and this preprint suggests that purifying selection pressure on the spike gene of SARS-CoV-2 may increase with increasing vaccination rate.^1^ Further study is clearly warranted to augment our collective understanding of the biology of SARS-CoV-2 and its interplay with the human immune system in support of effective countermeasure development.

The viral genetic data derived from this study, when combined with the epidemiological data, demonstrate possible transmission chains and provide new information as to how quickly a virus may begin to diverge in a contained, immunologically naïve population. Our results demonstrate that genetic variations can occur constantly and rapidly over a relatively short period and that these mutations may be useful for tracking transmission chains. This is important because as novel viruses such as SARS-CoV-2 evolve rapidly after infection the mutations may affect virulence, infectivity, and transmissibility. In addition, this study demonstrates how sequencing-based surveillance, whether it be targeted sequencing as in this study, or whether it be unbiased shotgun sequencing, can be used for molecular epidemiological purposes to support force health protection decision-making, such as to track and halt transmission chains with additional protective measures. Finally, our study confirms the need to accumulate more sequence data from this outbreak to better trace the viral genome evolution and associate the changes with epidemiological data and clinical symptoms. A better understanding of this, and other similarly isolated SARS-CoV-2 outbreaks, will aid in the preparation for containment of future shipboard outbreaks.

## Data availability statement

The datasets generated and analyzed for this study can be found in the NCBI GenBank at: https://www.ncbi.nlm.nih.gov/nuccore/, accession MW130903-MW130920.

## Author contributions

BP, EA, and MP-S performed sample collection and COVID-19 testing for shipboard surveillance. KS and DS performed virus culture. LG and FM performed genome sequencing. LV, RC, ML, EA, JR, and KB-L performed data analyses. RC, LV, BA, BP, and KB-L wrote the manuscript. All authors contributed to the article and approved the submitted version.

## Funding

This study was supported by the Armed Forces Health Surveillance Division (AFHSD), Global Emerging Infections Surveillance (GEIS) Branch, ProMIS IDs P0013_AH_01.01 to KB-L as well as Navy Work Unit Number (WUN) A1417.

## Conflict of interest

LV, ML, LG, and FM were employed by the company Leidos.

The remaining authors declare that the research was conducted in the absence of any commercial or financial relationships that could be construed as a potential conflict of interest.

## Publisher’s note

All claims expressed in this article are solely those of the authors and do not necessarily represent those of their affiliated organizations, or those of the publisher, the editors and the reviewers. Any product that may be evaluated in this article, or claim that may be made by its manufacturer, is not guaranteed or endorsed by the publisher.

## Author disclaimer

The views expressed in this article are those of the authors and do not necessarily reflect the official policy or position of the Department of Defense, Department of the Navy, nor the U.S. Government. Several of the authors are U.S. Government employees. This work was prepared as part of their official duties. Title 17 U.S.C. § 105 provides that “Copyright protection under this title is not available for any work of the United States Government.” Title 17 U.S.C. §101 defines a U.S. Government work as a work prepared by a military service member or employee of the U.S. Government as part of that person’s official duties.

## Footnotes

^1^BBTools User Guide.

^2^ https://doi.org/10.1101/2021.08.08.21261768
